# Mechanism of drug resistance in HIV-1 protease subtype C in the presence of Atazanavir

**DOI:** 10.1016/j.crstbi.2024.100132

**Published:** 2024-02-20

**Authors:** S.V. Sankaran, Sowmya R. Krishnan, Yasien Sayed, M. Michael Gromiha

**Affiliations:** a Department of Biotechnology, Bhupat and Jyoti Mehta School of Biosciences, Indian Institute of Technology Madras, Chennai, 600036, India; b Protein Structure-Function Research Unit, School of Molecular and Cell Biology, University of the Witwatersrand, Johannesburg, South Africa

**Keywords:** HIV-1 protease, Binding affinity, Molecular dynamics simulations, Subtype C, Drug resistance

## Abstract

AIDS is one of the deadliest diseases in the history of humankind caused by HIV. Despite the technological development, curtailing the viral infection inside human host still remains a challenge. Therapies such as HAART uses a combination of drugs to inhibit the viral activity. One of the important targets includes HIV protease and inhibiting its activity will minimize the production of mature structural proteins. However, the genetic diversity and the occurrence of drug resistant mutations adds complexity to effective drug design. In this study, we aimed at understanding the drug binding mechanism of one such subtype, namely subtype C and its insertion variant L38HL. We performed multiple molecular dynamics simulations along with binding free energy analysis of wild-type and L38HL bound to Atazanavir (ATV). From the analysis, we revealed that the insertion alters the hydrogen bond and hydrophobic interaction networks. The alterations in the interaction networks increase flexibility at the hinge-fulcrum interface. Further, the effects of these changes affect flap tip curling. Moreover, the changes in the hinge-fulcrum-cantilever interface alters the concerted motion of the functional regions leading to change in the direction of flap movement thus causing a subtle change in the active site volume. Additionally, formation of intramolecular hydrogen bonds in the ATV docked to L38HL restricted the movement of R1 and R2 groups thereby altering the interactions. Overall, the changes in the flexibility of flap together with the changes in the active site volume and compactness of the ligand provide insights for increased binding affinity of ATV with L38HL.

## Highlights

• The insertion variant L38HL in HIV protease alters the hydrogen bond and hydrophobic interaction networks.
• It increases the flexibility at the hinge-fulcrum interface and affects flap tip curling.
• Insertion changes the direction of flap movement and the active site volume.
• Changes in the flexibility of flap along with the active site volume and compactness of the ligand explains the increased binding affinity of atazanavir with L38HL.

## Graphical abstract

**Figure:**
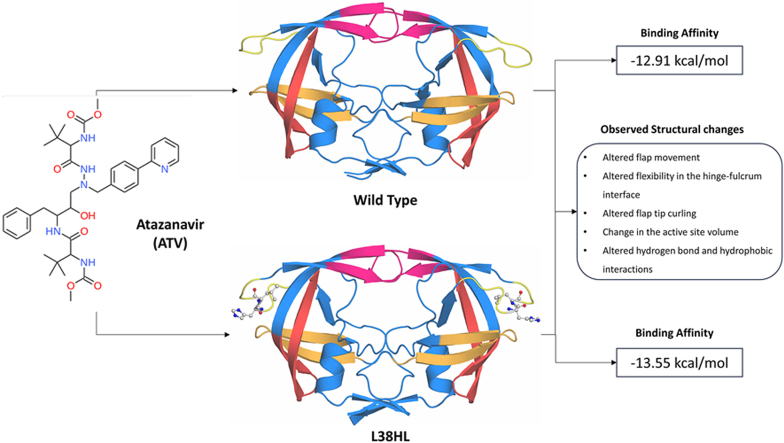

## 1 Introduction

Acquired Immunodeficiency syndrome (AIDS) is one of the deadliest and most devastating diseases in the history of humankind, which is caused by the Human Immunodeficiency Virus (HIV). HIV enters the host cell and affects the immune system (Barré-Sinoussi et al., 1983; Gallo et al., 1983). Approximately 40 million people are infected with HIV with 650,000 deaths per year, and most of them (68.8%) are from South African regions. (World Health Organization, 2021). In 1985, FDA approved its first drug, Azidothymidine (AZT) for treating HIV-1 infections (Dakshinamoorthy et al., 2023). Nevertheless, mitigating this viral infection is still a challenge. Current strategies involving effective drug design focus on three essential enzymes; namely, protease, reverse transcriptase, and integrase (Tözsér, 2003). Among the HIV proteins, protease has gained much attention due to its functional importance.

HIV protease (HIV PR) is responsible for the maturation of the HIV virion. It cleaves Gag and Gag-pol polyproteins, producing matured viral particles (Konvalinka et al., 2015; Weber et al., 2021). HIV PR is an aspartic protease belonging to a class of enzymes with conserved Asp-Thr/Ser-Gly residues in their active site. It is a homodimer, with each monomer comprising 99 amino acids and the dimer interface constitutes the active site (Copeland and Oroszlan, 1988; Lapatto et al., 1989; Navia et al., 1989). In addition to the active site, the HIV PR has other functionally important regions; namely flap (residues 46–54), hinge (residues 35–42), fulcrum (residues 10–22), and cantilever (residues 62–78). The activity of HIV PR is dependent on the concerted movement of these functionally important regions. This coordinated movement aids in the opening and closing of the flap region, which is crucial for enzyme function (Sherry et al., 2021). Thus, inhibiting the enzyme activity is achieved by arresting the to-and-fro movement of the flap. Till date, FDA has approved ten protease inhibitors (PIs), namely; saquinavir (SQV), ritonavir (RTV), tipranavir (TPV), indinavir (IDV), atazanavir (ATV), fosamprenavir (FPV), amprenavir (APV), nelfinavir (NFV), lopinavir (LPV), and darunavir (DRV) (Dakshinamoorthy et al., 2023; Wensing et al., 2010). All these inhibitors are designed to target the active site of the enzyme. Thus, any changes in the residues lining the active site will have a direct impact on drug susceptibility. For instance, the G48V active site mutation induces a conformational change due to a steric clash with SQV, thereby affecting its binding (Wittayanarakul et al., 2005) In addition, mutations that occur away from the active site are also known to induce drug resistance (Weber et al., 2021).

**Fig. 1:**
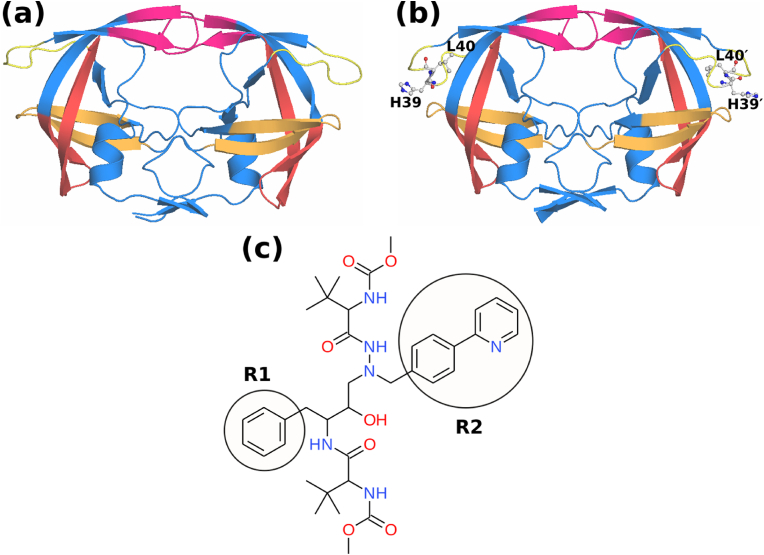
3D structure of HIV protease subtype C (a) wild-type and (b) L38HL variant. The structures are colored based on the functional regions namely; flap (magenta), cantilever (red), fulcrum (orange) and hinge (yellow). The insertion of residues at the hinge in L38HL is shown in ball-and-sticks and labeled. (c) 2D structure of Atazanavir (ATV).

It is important to note that all the available drugs are designed and effective mainly for HIV-1 PR subtype B. Specifically, inhibitors such as lopinavir, ritonavir, saquinavir, indinavir, and amprenavir exhibited decreased affinity for subtype C (Eche et al., 2021; Mosebi et al., 2008; Shafer et al., 1999; van Zyl and Decloedt, 2016; Velázquez-Campoy et al., 2003), characterized by eight distinct non-active site mutations (Naicker et al., 2013). Furthermore, several primary and secondary mutations in the HIV-1 PR subtype C have also been reported. Recently, we identified one such variant (L38HL), characterized by the insertion of two residues histidine and leucine after L38 (the sequence 38LP39 is changed to 38LHLP41; the inserted residues are underlined) (Ledwaba et al., 2019). The 3D structures of HIV-1 PR subtype C wild-type (WT) and the L38HL variant are shown in Fig. 1. Understanding the effect of such insertions on the overall dynamics is of utmost importance to designing potent inhibitors targeting HIV infections.

In this work, molecular dynamics simulation of the WT and L38HL variant in the presence of a second-generation antiretroviral PI atazanavir (ATV) was performed. Results from the MD simulations and binding free energy analysis showed that the L38HL variant displayed a moderate increase in the binding affinity for ATV with a difference of 0.64 kcal/mol. Further, analysis of the local structural changes revealed an increase in active site volume and flap tip curling. Additionally, an inspection of the inter-and intra-molecular changes showed that several contacts are lost at the hinge-fulcrum interface.

## 2 Methods

### 2.1 Protein structure preparation

The structures for the closed conformation of wild-type and L38HL of HIV protease subtype C are obtained through homology modeling using MODELLER (Webb and Sali, 2016) based on the structure of HIV protease subtype B in closed conformation as template (PDB code: 2AQU). The modeled structures were then docked with the ATV using AutoDock (Forli et al., 2016). The conformation with the lowest binding energy was chosen for further analysis. The docked structures were then used as the initial co-ordinates for MD simulations.

### 2.2 System preparation

All the simulations in this study were performed using AMBER18 (Case et al., 2005). The topology and the co-ordinate files were obtained using the LEap module of AmberTools with AMBER ff14SB (Maier et al., 2015) force field parameters. The ATV molecule was parameterized with the Antechamber module of AmberTools using the General Amber Force Field (GAFF) force field (Wang et al., 2004). The system was solvated with TIP3P water molecules and neutralized by adding Cl^−^ counter ions in a cubic box with at least 1 nm distance between the box and the protein surface.

Initially, protein atoms were restrained with the force constant of 100 kcal mol^−1^Å^−1^ followed by minimization of the entire system in the subsequent minimization steps. The minimization steps were carried out for a maximum of 1500 iterations, with 500 steps of steepest descent and 1000 steps of conjugate gradient. Next, the systems were allowed to transit from 10 to 300 K by restraining the protein atoms through 1 ns of equilibration. A Langevin thermostat with constant periodic boundary conditions was applied. Further, the restraints were removed, and the system was equilibrated at 300 K through 4 ns of MD simulation. All simulations were performed with an integrator step of 2 fs, and trajectories were written for every ps. The system was treated under periodic boundary conditions, and electrostatics was treated using Particle Mesh Ewald (PME) (Darden et al., 1993) with a cut-off of 10 Å for non-boned interactions. Finally, for each of the starting structures (WT and L38HL), three independent production runs of 300 ns each were performed by applying different initial velocities.

### 2.3 MD simulation analyses

The trajectories were analyzed using the CPPTRAJ module of AmberTools. The root-mean-squared deviation (RMSD) was calculated by aligning the C-alpha atoms of the protein in each snapshot to the initial structure. The root-mean-square fluctuation (RMSF) values for each residue with respect to the initial structure were obtained by performing an RMS fit of all protein atoms except hydrogens to the initial structure. All the analyses, unless specifically mentioned, were performed for the last 250 ns of the trajectories. Further, all the calculations and quantitation presented in this study correspond to the average of three runs.

### 2.4 Hydrogen bond analysis

The hydrogen bonds in the WT and L38HL systems were determined using the CPPTRAJ module of the AmberTools*.* The frequency values for each of the atom pairs were calculated with a donor-acceptor (D-A) distance cut-off of 3 Å and an angle cut-off of 135° (D-H-A). Further, the average 2D hydrogen bond map was constructed for the WT and L38HL systems using in-house Python scripts and plotted using MATLAB (*MATLAB Version 9.13.0.2126072 (R2022b)*, 2023).

#### 2.4.1 Hydrogen bond maps

The axes in the hydrogen bond maps represent the residue number. A dot on the map depicts a hydrogen bond interaction between the corresponding residues. The residue-wise hydrogen bond information was obtained through the summation of frequency values of the atom pairs belonging to the residue pairs (Badaya and Sasidhar, 2020). For example, if one of the atoms of Arg57 forms a hydrogen bond with any of the atoms of Glu35 and vice versa, the frequency values from all of the atom pairs are added to get the total hydrogen bonding frequency between Arg57 and Glu35.

### 2.5 Hydrophobic contacts

In view of understanding the changes in the hydrophobic interactions, the residue-wise hydrophobic contacts were assessed in the WT and L38HL systems. The criteria used for calculating hydrophobic contacts are as follows. If any one atom of hydrophobic residue A is within or equal to 5.0 Å of any other atom of hydrophobic residue B, then residues A and B are said to be in hydrophobic contact with each other. The average frequency of each residue pair over the period of simulations is presented as a hydrophobic contact map. All the calculations were performed using an in-house Python script.

### 2.6 Molecular mechanics Poisson-Boltzmann surface area calculation

The binding free energy of ATV with WT and L38HL systems was computed using the Molecular Mechanics Poisson-Boltzmann surface area (MM-PBSA) method (Miller et al., 2012). All the calculations were performed using the MM-PBSA module from the AmberTools. For each binding complex, 1000 snapshots from the last 100 ns of the trajectory with an interval of 100 ps were taken for analysis. The binding free energy of the complexes is calculated as shown in Equation (1),

(1) $$\Delta G_{bind}=G_{Complex}-G_{receptor}-G_{ligand}$$

Where, $\Delta G_{bind}$ is the change in binding free energy, $G_{Complex}$ , $G_{receptor}$ , $G_{ligand}$ are the binding free energies of complex, receptor, ligand respectively. The binding energy $\left(\Delta G_{bind}\right)$ is calculated from the sum of enthalpy (ΔΗ) and the entropy (ΔS) components as mentioned in Equation (2). The enthalpy, on the other hand, is calculated by summing the electrostatics $\left(\Delta E_{elec}\right)$, van der Waals $\left(\Delta E_{vdw}\right)$, polar solvation $\left(\Delta G_{pb}\right)$ and non-polar solvation $\left(\Delta G_{SA}\right)$ terms as shown in Equation (3). Further, the polar solvation term is calculated using the Poisson-Boltzmann equation and the non-polar solvation term is calculated using an empirical method based on solvent-accessible surface area (ΔSASA) (Miller et al., 2012)

(2) $$\Delta G_{bind}=\Delta Η-Τ\Delta S$$

(3) $$\Delta Η=\Delta E_{elec}+\Delta E_{vdw}+\Delta G_{pb}+\Delta G_{SA}$$

(4) $$\Delta G_{SA}=\gamma \Delta SASA+\beta$$

The default values of γ and β were used for the empirical calculations. The dielectric constant of the solute and solvent was set to 4.0 and 80.0, respectively (Wang et al., 2020; Wang and Zheng, 2020).

## 3 Results

### 3.1 Assessing the flexibility and compactness of WT and L38HL complexes

**Fig. 2:**
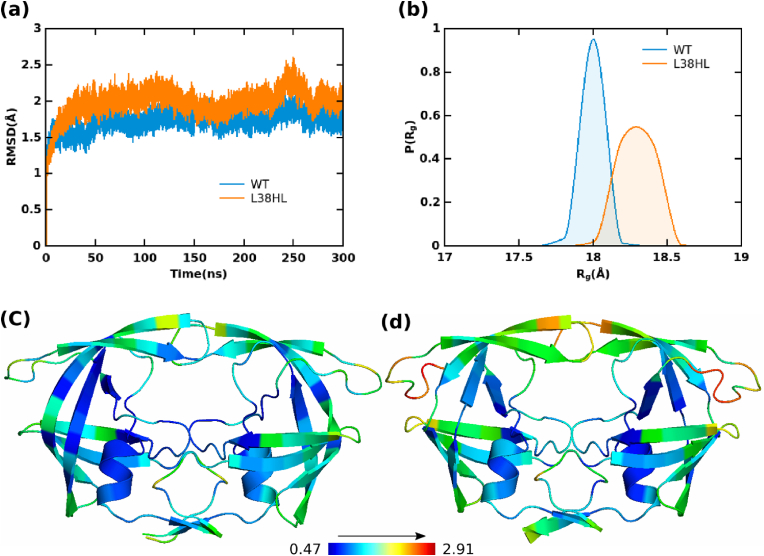
(a) Time evolution of average C-alpha RMSD for WT and L38HL systems; (b) probability distribution of radius of gyration from (R_g_) the MD data showcasing the compactness of the PR-inhibitor complexes; (c) mean RMSF from the MD simulation mapped on the structures of WT and (d) L38HL. The RMSF values are scaled based on the minimum and maximum (values mentioned in the color bar) from the mean RMSF of both the WT and L38HL. The color bar is in the unit of Å. Regions of the HIV-1 PR colored in blue represent residues with low fluctuation while those in the red represent higher fluctuation.

To gain insights into the conformational flexibility due to the insertion of residues in the HIV-1 PR subtype C bound to Atazanavir (ATV), all-atom MD simulations of WT and L38HL with ATV were performed. The conformational stability of these complexes was assessed by computing root-mean-square deviation (RMSD). The time evolution of mean RMSD computed from three replicas for WT and L38HL is shown in Fig. 2a. As can be seen from Fig. 2a, both systems were stable throughout the simulation period, and no large-scale conformational changes were observed. The RMSD values vary between 0.8 and 1.8 Å for WT and 1.5 and 3.5 Å for L38HL. The difference in the range of mean RMSD is due to the subtle structural re-arrangements that arise due to the insertion of residues. In general, the mean RMSD in both the trajectories achieves equilibrium after ∼50 ns. Thus, the initial 50 ns of the trajectories were not considered, and the analyses, unless specified, were performed for the last 250 ns of the MD trajectories.

The radius of gyration (R_g_) calculations showed deviations between the WT and L38HL, as revealed by significant changes in the R_g_ distributions (Fig. 2b). The R_g_ values range between 17.6 and 18.2 Å for WT and 18 and 18.6 Å for L38HL. The peak depicting the more probable conformations is located around 18 Å and 18.4 Å for WT and L38HL, respectively. The mean and standard deviation (SD) of WT is 17.8 Å and 0.049 Å, and that of L38HL are 18.2 Å and 0.095 Å, respectively. Further, it is to be noted that there is no considerable overlap between the WT and L38HL trajectories, suggesting that both WT and L38HL complexes sample distinct conformations. Moreover, WT samples have less conformational change due to their compact nature, while L38HL samples more conformations.

Additionally, the results from the solvent-accessible surface area (SASA) analysis agree well with the radius of gyration results (Fig. S1). The distribution of SASA depicts the overall increase in the solvent-accessible nature of the L38HL system. Moreover, the difference in solvent accessibility is more pronounced in the active site region (Fig. S1). This led us to conclude that the insertion of residues in the hinge region of the PR complex could increase its overall flexibility.

### 3.2 Insertion at the hinge induces the overall fluctuation of L38HL

In order to understand the change in fluctuations at the residue level, the root-mean-square fluctuation (RMSF) of all the atoms excluding hydrogens was computed. The computed mean RMSF values from three independent simulations were mapped onto the structures. Regions with low fluctuations are colored blue, and regions with high fluctuations are colored red. Fig. 2c and d represent the mean RMSF fluctuation of WT and L38HL residues, respectively. The flexibility patterns obtained from the WT were different from L38HL. As can be seen from Fig. 2c and d, the hinge region of L38HL, where the insertions occur, exhibits maximum fluctuation with a mean RMSF value of ∼2.91 Å. Apart from the hinge, the flap and the fulcrum regions of L38HL showed higher mobility. As the movement of the flap is strongly implicated in maintenance of the network of hydrogen bonds and hydrophobic interactions between the hinge, fulcrum and the cantilever, it can be expected that the increased mobility of the hinge may have a direct influence on the flexibility of the fulcrum and the flap regions. Also, it should be noted that in HIV-1 PR, the residues lining the catalytic site are expected to exhibit a high degree of rigidity. However, it was observed that the active site residues of the L38HL were comparatively more flexible than the corresponding active site residues in the WT protease. Thus, the overall increase in the RMSF of L38HL clearly explains the increased conformational flexibility due to the subtle conformational changes leading to an altered network of hydrogen bonds and hydrophobic interactions associated with the insertion.

### 3.3 Weakening of hydrogen bond interactions in L38HL affects concerted motion owing to increased flap flexibility

**Fig. 3:**
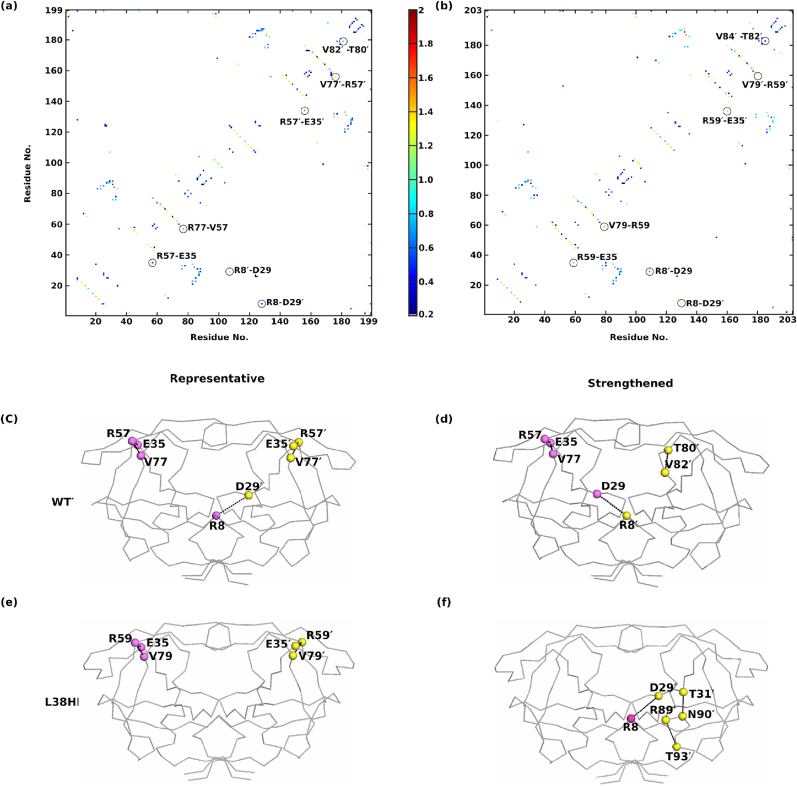
Residue-wise average hydrogen bond contact map of (a) WT and (b) L38HL. Each dot on the map represents that a hydrogen bond is formed between those residues. Further, the color bar obtained from the frequency of frames is used to quantify the strength of the hydrogen bonds. Only contacts having frequency greater than or equal to 0.2 are shown. The representative position of hydrogen bonds is mapped onto the structure of (c) WT and (d) L38HL. The hydrogen bonds that are strengthened in (e) WT and (f) L38HL. The protein backbone is shown in ribbon and the residues are represented based on the C-alpha atoms. Other residues are not shown for clarity. Atoms corresponding to chain A are colored violet and those corresponding to chain B are colored yellow.

The results from the RMSF (section 3.1) and R_g_ (section 3.2) analyses imply that insertion increases flexibility of the hinge, fulcrum and flap regions. The plausible explanation for the increased mobility can be the altered network of interactions. In particular, the insertion of H and L at the hinge region is expected to modify the structural packing and, hence, the hydrogen bond network. To understand this quantitatively, mean residue-residue hydrogen bonding contact maps from three independent simulations for WT and L38HL were generated. Further, to investigate the impact of hydrogen bonding in WT and L38HL, the frequency values of WT from L38HL were subtracted. Thus, the residue pairs with higher frequency in the WT are designated as strengthened in WT, and those with higher frequency in L38HL are considered to be strengthened in L38HL. A detailed description of the calculations and contact map generation is given in the Methods (section 2.4). The hydrogen bonding maps for WT and L38HL and their corresponding position in the structure are shown in Fig. 3.

**Fig. 4:**
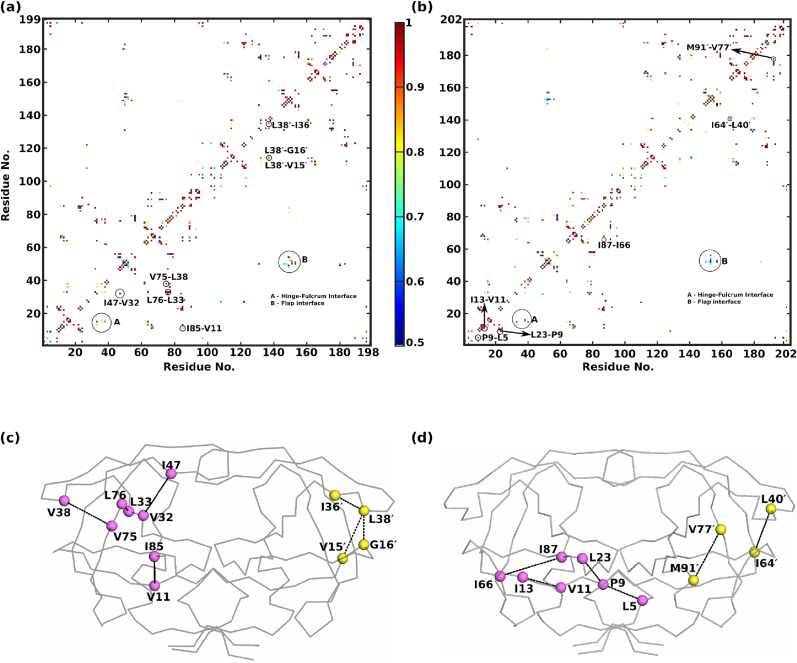
Contact maps showing the average hydrophobic interaction (a) WT, (b) L38HL. Each dot on the contact map represents the formation of a hydrophobic interaction between a pair of protein residues. Continuous numbering is followed for clarity. The contacts that are strengthened are mapped onto the 3D structure (c) WT, (d) L38HL. Protein backbone is represented in the ribbon structure and the residues are represented by C-alpha atoms. Atoms corresponding to chain A are colored violet and chain B are colored yellow. Other residues are not shown for clarity.

Overall, most of the hydrogen bonding residue pairs in WT were conserved in L38HL; nevertheless, some minor yet critical differences in the contact maps were observed. From Fig. 4a the frequency of hydrogen bonds formed by residue pairs R57-E35, has decreased significantly in L38HL. However, in the case of R57′-E35′, the difference in the frequency values was less compared to the other subunit. Additionally, the hydrogen bond frequency of R57-V77 was also decreased in L38HL. These results are consistent with a previous study (Naicker et al., 2013), which substantiates the role of the R57-E35 salt bridge in flap flexibility.

Further, the residue-pair T80′- V82′ appeared to be strengthened in WT protease. On the other hand, residue pairs R89′-T93′ and T31′-N90′ were strengthened in L38HL. An interesting pattern in the hydrogen bond strength was observed in residue pairs R8′-D29 and D29′-R8, where the former was strengthened in WT and the latter in L38HL. Given the hydrogen bonding contact maps, it can be inferred that the insertion at the hinge both promotes as well as hinders hydrogen bond formation. From the position of these residues (Fig. 3c–f), it can be understood that the insertion weakens hydrogen bonds between the inner β-strand and flap, and at the hinge-fulcrum interface. While it strengthens at the fulcrum-cantilever interface of chain B. This weakening of hydrogen bonds in L38HL disrupts the concerted movement of flaps and hinges, making it more flexible. Also, it is essential to note that the hydrogen bonds strengthened in WT protease stabilize both the monomers, while in L38HL, those belonging to chain B are strengthened.

### 3.4 Hydrophobic contact analyses

To investigate the perturbations induced by insertions on the hydrophobic interactions, hydrophobic contacts of WT and L38HL were examined. A detailed description of the calculation is given in the Methods section. A comparison of the average hydrophobic contact maps obtained from the calculations is presented in Fig. 4. Only residue pairs with contact frequency ≥0.5 are shown.

Despite a few changes in the contact strengths and interaction between residue pairs, the interaction pattern of L38HL closely resembles WT. Particularly, the changes in the interaction patterns were observed at the hinge fulcrum interface. The result demonstrates that the insertion at the hinge alters the interaction with the fulcrum, which influences the interactions at the flap. Additionally, we evaluated the strength of contacts in WT and L38HL. The residue pairs I47–V32, V75-L38, L76-L33, I85–V11, L38′-V15′, L38′-G16′, and L38′-I36′ are strengthened in WT; in other words, these contacts are weakened in the L8HL system. Importantly, all these residue pairs that are weakened in L38HL involve residues adjacent to the position of insertion. On the contrary, the contacts between residues I13–V11, P9-L5, L23-P9, I87–I66, I64′-L40′, and M91′-V77′ are strengthened in L38HL. The contact strength (quantified by the frequency of frames) of I47–V32, V75-L38, and L76-L33 in WT is 0.96, 0.97, 0.93, 0.75 respectively, whereas it reduced to 0.67, 0.72, 0.76 and 0.51, respectively, in L38HL. Similarly, the strength of contacts between the residue pairs L38′-V15′, L38′-G16′, and L38-I36 are 0.97, 0.86, and 0.99 in WT whereas, respectively, while in L38HL, they reduced to 0.68, 0.59, 0.45, respectively.

Further, to unearth the role of strengthened contacts in the PR dynamics, the position of these contacts was mapped onto the structures, and the same is presented in Fig. 4c and d. Upon comparing the location of the contacts that are strengthened in WT, we suggest that these contacts help to achieve the concerted movement of the flap with the fulcrum, hinge, and cantilever, whereas weakening these contacts in L38HL affects the concerted motion owing to increased flexibility. On the other hand, except for L40′-I64′, the location of strengthened residue pairs in L38HL suggests that the contacts are strengthened only within the fulcrum and N-terminal residues.

The results from hydrophobic interaction analysis correlate well with the RMSF results, where it is established that the fluctuations at the hinge and the flap regions are more in L38HL. This leads us to conclude that the insertion at the hinge causes perturbations in the network of hydrophobic interactions at the hinge region. In turn, these changes cause weakening of interaction of the hinge with the fulcrum and flap regions owing to loss of concerted motions in L38HL. Further, the weakening of interactions in L38HL may be related to the overall flexibility of the PR, making it less compact than WT. These explanations are consistent with the results from the radius of gyration.

### 3.5 Analyzing the differences in local fluctuations of WT and L38HL

Studies have shown that both major/primary mutations (in the active site region) and minor/secondary mutations (away from the active site) cause local structural changes. For instance, in our previous work, we reported that the distance between the Val32 and Val32’ increased in the L38HL-SQV complex (Venkatachalam et al., 2023). Moreover, in HIV-1 PR subtype B, mutations such as L90M and V82F cause a wide opening of the flap compared to the WT (Maschera et al., 1996; Perryman et al., 2004). Therefore, to reveal the structural changes happening at the local level, we investigated the local fluctuations, including the distance between the active site and the flap tips, and the flap tip tri C-alpha angle in WT and L38HL (R. Wang and Zheng, 2020; Yu et al., 2021).

#### 3.5.1 Distance between the flap tips and the active site

**Fig. 5:**
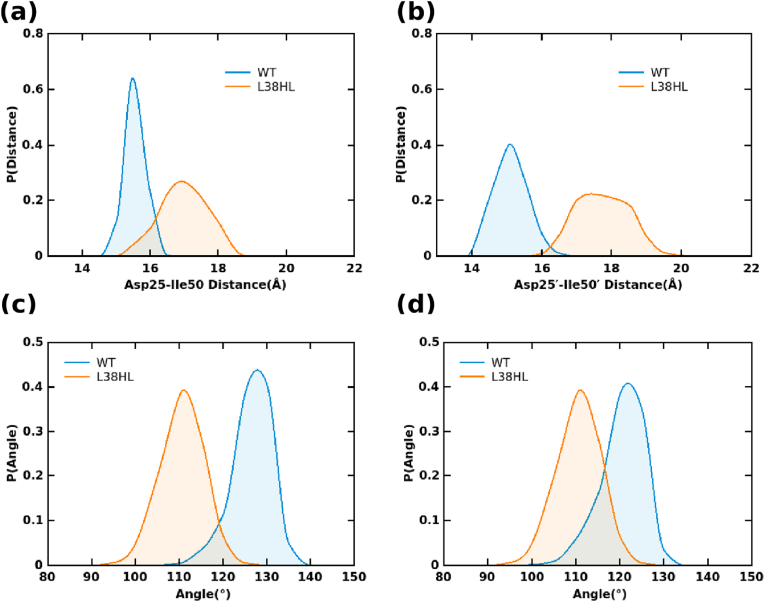
Average distance distribution of active site to flap distance of WT and L38HL systems corresponding to (a) chain A and (b) chain B. Average angle distribution of tri C-alpha angles from flap tips corresponding to (c) chain A, and (d) chain B.

To understand the relative changes of the flap with the active site, the distance between the flap tips characterized by C-alpha atoms of Ile50/Ile50′ for WT Ile51/Ile52′ for L38HL, and C-alpha atoms of active site residues Asp25/Asp25’ were calculated (Wang et al., 2020; Wang and Zheng, 2020; Yu et al., 2021). To precisely capture the differences that occur in both the chains, the calculations were performed separately for each of the chains. The distribution obtained from the average distance values of three replica simulations is shown in Fig. 5a and b.

Both WT and L38HL showed a single peak distribution. For WT, the peak is around 15 Å for chain A and chain B, while for L38HL, the peak is around 17 Å for chain A and 17–17.5 Å for chain B. The average distances between the flap tips and the active site are 15.30 ± 0.26 Å, 14.85 ± 0.47 Å, 16.73 ± 0.68, and 17.49 ± 0.74 for WT chain A, WT chain B, L38HL chain A and L38HL chain B, respectively. Moreover, the observation of the area under the curve depicts that L38HL exhibits diverse conformational sampling compared to WT. These results are consistent with the previous studies (Wang et al., 2020; R. Wang and Zheng, 2020; Yu et al., 2021). The differences in the average distribution clearly denote that the distance between the flap tips and the active site is smaller in WT than in L38HL. Further, the differences in the average values highlight that, despite the homodimer nature, each chain exhibits a different dynamic behavior. These changes in L38HL could probably affect the interactions of ATV with PR.

#### 3.5.2 Analysis of tri C-alpha angles

To derive specific inferences on the influence of flap tips on the increased flap-active site distances, the tri C-alpha angles corresponding to flap tips (G49/G51′-I50/I51′-G51/G53′) were calculated. The distribution of flap tri C-alpha angles is shown in Fig. 5c and d. It can be seen that the distribution corresponding to chain A shows minimal overlap between the WT and L38HL, while in the case of chain B, they overlap partially. The angles sampled in chain A in WT range from ∼110° to ∼140° with a peak around 125° and from ∼100° to ∼120° with a peak around 110° for L38HL. Similarly, the angles sampled by chain B in WT are in the range of ∼110°–∼130° with a peak around 125° for WT and ∼100–∼120° with a peak around 110° for L38HL. The average flap tri C-alpha angles are 123.97 ± 4.35°, 122.97 ± 4.80°, 119.68 ± 4.21°, 113.04 ± 4.97° for WT chain A, WT chain B, L38HL chain A and L38HL chain B, respectively. Hence, the differences in the average angle values imply that the event of flap curling is more pronounced in L38HL than in WT.

The results from the active site flap tip distance distributions and the tri C-alpha flap tip angle distribution can be associated with the hydrogen bond and hydrophobic analyses. The changes in the hydrophobic interaction pattern at the flap interface of L38HL agree well with the flap curling, demonstrating that the insertion of residues in the hinge causes weakening of interactions between the hinge and fulcrum. In turn, it can be expected that these changes alter the flap dynamics, and one such event is the flap curling.

### 3.6 Altered dynamics of the WT and L38HL flaps

To derive the generalized inferences on the collective, concerted motion of the residues in the WT and L38HL, the essential dynamics (ED) was performed. The motion of the residues is characterized by eigenvalues and eigenvectors of the positional covariance matrix (Venkatachalam et al., 2023), in which the eigenvalues represent the motion along the eigenvectors. The direction of motion of the residues in WT and L38HL were obtained using the porcupine plots. The length of the porcupine represents the amplitude of motion, and the porcupine represents the direction of motion of C-alpha residues. The porcupine plot corresponding to all three replicas from WT and L38HL are presented in Fig. S2. In all the replicas corresponding to WT and L38HL, the maximum contributions come from the first principal component. Therefore, only the plots corresponding to the first principal component are shown. It is evident from the porcupine plots that the dynamics of the entire protein is altered in the L38HL as a result of the insertion. Specifically, significant changes in the direction of motion are observed in the hinge and the flaps, while the changes were less pronounced in the active site regions. In the case of WT, the residues from the flap show the tendency to move towards the active site, allowing them to come closer, while in the case of L38HL, the flap residues tend to move away from the active site in a manner that could lead to flap opening. Except for the hinge region, both WT and L38HL showed similar amplitude of motion. The results from ED ascertain our previous results. Thus, the changes at the hinge significantly alter the flap motions of the L38HL system.

### 3.7 Intramolecular hydrogen bonding in ATV

**Fig. 6:**
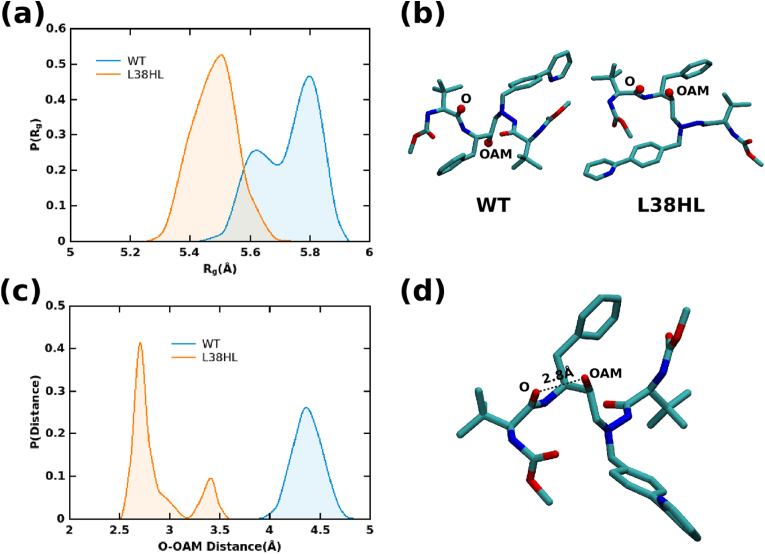
Radius of gyration of ligand in (a) WT and L38HL. (b) Representative conformation of the ligand is shown with the positions of O and OAM. Left panel corresponds to the WT and right panel corresponds to the L38HL variant. (c) The atoms O and OAM are represented as red spheres. The density distribution of distance between atoms O and OAM. (d) Representative conformation of ATV showing the formation of a hydrogen bond between O and OAM atoms.

Apart from understanding the conformational changes in the protein, the conformational changes in the ligand were investigated. Interestingly, an intramolecular hydrogen bond that is formed between the atoms OAM and O (Fig. 6), in which the O accepts a hydrogen atom from the OAM, is observed with ∼90% occupancy in all three replica simulations of L38HL. We further computed the histogram distribution of distance between O and OAM. From Fig. 6c, it can be observed that the distance between O and OAM is predominantly between 2 and 3 Å with an additional, albeit, smaller peak at ∼3.4 Å for L38HL, and for WT, the distance ranges from 4 Å to 4.6 Å. The occurrence of the second peak in L38HL (3–3.5 Å) is likely because of the conformations sampled in the remaining 10% occupancy. The results from hydrogen bond and distance calculations establish the formation of intramolecular hydrogen bonds in ATV corresponding to L38HL. The formation of such hydrogen bonds will significantly influence the ligand conformational sampling, which, in turn, can alter its compactness.

We further examined the compactness of ATV in both systems by computing the radius of gyration. The average histogram distributions of WT and L38HL systems (Fig. 6a) were obtained from the individual replica simulations. As can be seen from Fig. 6, ATV in L38HL is more compact than ATV in WT. Moreover, the distribution corresponding to WT has two peaks at ∼5.6 Å and ∼5.8 Å, with the latter one having a higher sampling frequency than the former. The occurrence of bimodal distribution implies that the ligand has sampled two different conformational states. On the other hand, the distribution corresponding to ATV in L38HL is unimodal, with a peak value of ∼5.5 Å.

**Fig. 7:**
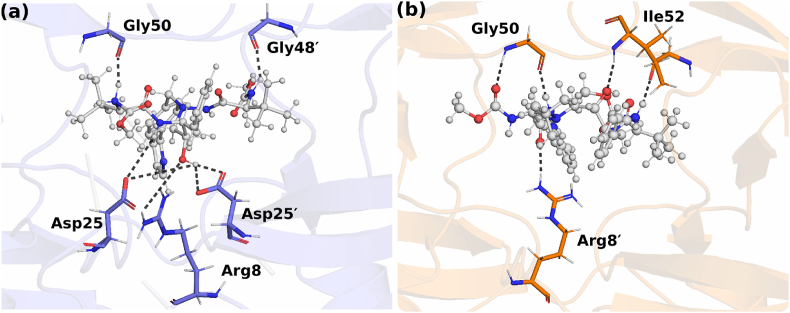
Representative structure showing the hydrogen bond interaction of (a) WT and (b) L38HL with ATV. ATV is represented in ball and sticks for the ease of visualization.

Overall, the presence of hydrogen bonds between the OAM and O in ATV restricts the movement of the R1 and R2 groups (Fig. 6 b). This conformational restriction in the ATV alters its interaction pattern with WT and L38HL. As can be seen in Fig. 7, the atoms of ATV establish hydrogen bond interactions with the WT active site residues, whereas, the interactions with the active site residues are hindered in L38HL with more interactions established with the flap residues. Additionally, the torsion angle calculated (Fig. S3) between atoms NBG and CBT (C-NBG-CBT-CBS) showed significant differences in the distribution of ATV in WT and L38HL.

### 3.8 Binding free energy analysis of WT and L38HL complexes

To comprehend the differences in the binding of ATV in WT and L38HL, the binding free energy analysis was performed using the MM-PBSA module of AMBER. The analyses were performed for 1000 frames obtained using a constant interval from the equilibrated trajectory. A detailed description of the calculation is given in the Methods section.

The average binding free energy of WT and L38HL is −12.91 (kcal/mol) and −13.55 (kcal/mol), respectively, with L38HL having a slightly higher value than the WT. The difference in the binding energy is 0.64 kcal/mol. The subtle change in the binding energy is a result of the change in the interaction pattern and altered dynamics at the flap region. As can be seen, the changes in the interaction come from the polar solvation terms, indicating that the ligand is more exposed to the solvent in WT. This can be associated with the formation of hydrogen bonds in the ATV that restrict its movement and help to attain compactness.

## 4 Discussion

The insertion of two residues in the hinge region has significantly altered the dynamics of HIV-1 PR subtype C. Previous studies on HIV-1 have shown that the non-active site mutation in the PR has greatly influenced drug susceptibility (Bastys et al., 2020; Dakshinamoorthy et al., 2023). Despite the fact that the structural changes caused by these mutations are minimally reflected in the active site, such changes will alter the overall dynamics of the PR enzyme, thereby affecting drug binding (Sherry et al., 2021; Venkatachalam et al., 2023). Thus, we suggest that the conformational flexibility of the PR is crucial for its activity.

In agreement with the previously reported findings on HIV-1 PR (Venkatachalam et al., 2023), our results show that the insertion at the hinge has a considerable impact on its overall dynamics. It is noteworthy that the region with the insertion showed increased RMSF compared to its wild-type counterpart. The change in the RMSF is because of the perturbations in the network of contacts due to the insertion. One of the consistent observations is the loss of contact strengths between the hinge flap region and the fulcrum. Changes in the contact strength will disturb the concerted motion of the hinge and the fulcrum. Our results from the hydrogen bond analysis show the weakening of the contact between R58-E35 and R58–V77 in L38HL. Further, the hydrophobic contact analysis reported the weakening of contacts between the fulcrum, hinge, and flaps.

The distributions of flap tip angles and the relative changes of flap tips with the active site provide insights into the effect of changes in the hinge and fulcrum on the flap dynamics. The drastic difference in the distance and angle distribution is the consequence that arises due to the altered flap dynamics. Specifically, the patterns from the angle distributions of WT and L38HL indicate that the former remains in the closed state, while the latter exhibits curling and tends to move outwards.

Moreover, the directionality of the motion of residues, as observed from the ED, explicitly indicates that in addition to flap curling, the flap moves away from the active site, depicting the opening motion. The changes in the direction of flap movement ascertain our understanding that small structural changes can effectively alter the PR dynamics. Such changes in the flap can alter the binding site volume, which is observed from the increased SASA. Further, the movement of the flap away in the outward direction, together with the flap curling, leads to the increased binding site volume.

Discussing the effect of insertions on the ligand dynamics, it is interesting to observe the formation of intramolecular hydrogen bonding in ATV when bound to L38HL. Given this information, it can be understood that the formation of a stronger hydrogen bond restricts the sampling of R1 and R2 groups, thereby helping to achieve compactness. Further, the changes in the intramolecular hydrogen bonding induces changes in the hydrogen bonding interaction of ATV with the PR.

Overall, the insertion at the hinge region increases the flexibility of the HIV-1 PR subtype C by disrupting the concerted motion of the enzyme. Specifically, it induces altered dynamics of the flap, leading to increased active site volume, which could possibly be the result of allostery. Generally, allostery can be viewed as structural change at a site that is often the result of stimulus at another site. In this regard, the insertion at the hinge region can be attributed to the stimulus that has triggered several structural perturbations including flap curling, flap movement, which could have led to the increased active site volume. Unraveling such allosteric changes will increase our understanding about HIV-1 PR thereby leading to the development of drugs that completely mitigate the HIV-1 PR enzyme activity (Hertig et al., 2016; Nussinov and Tsai, 2013; Berezovsky and Nussinov, 2022).

On the other hand, the formation of intramolecular molecular hydrogen bonds between OAM and O atoms of ATV help to attain a compact structure. The formation of intramolecular hydrogen bond in ATV reduces its entropy in the L38HL-ATV complex. Further, the slight increase in the active site volume due to local conformational changes may establish hydrophobic and van der Waals interactions resulting in the gain of enthalpy. Hence, the loss of entropy is compensated by the enthalpic gain. Moreover, in the case of wild-type, ATV forms hydrogen bonds with active site residues whereas in L38HL these interactions are observed with the flap residues, which favors conformational changes near the active site region. Hence, we suggest that the combination of increase in the volume and the intramolecular hydrogen bond formation increase the binding affinity for L38HL-ATV complex. It is convincing to believe this notion because previous studies have shown that the mutation L76V mutation has decreased susceptibility for one SQV whereas it showed increased susceptibility for ATV.

## 5 Conclusions

HIV-1 protease is one of the instrumental targets for mitigating the HIV infection. Currently, there are 10 FDA-approved anti-retroviral drugs that are used to target HIV protease. Nevertheless, the potency of these drugs is hindered by the primary and secondary mutations in HIV-1 PR. One such variant is the subtype C, which is widely reported in the sub-Saharan regions and African countries. In this study, we used molecular dynamics simulations to understand the differences in interaction patterns of HIV-1 PR subtype C wild-type and an insertion variant L38HL, characterized by the insertion at the hinge region with the second-generation antiretroviral drug atazanavir (ATV). The insertion causes a perturbation in the PR interaction network, causing altered dynamics. Further, the change in the dynamics influences the ATV interaction with a slight increase in the hydrogen bonds in the WT-ATV complex compared to the L38HL-ATV complex. We conclude that the change in the active site volume along with the hydrogen bond in ATV, leads to a slight increase in the binding affinity of ATV in L38HL.

## CRediT authorship contribution statement

**S.V. Sankaran:** Data curation, Methodology, Computation, Validation, Formal analysis, Visualization, Writing – original draft, Writing – review & editing. **Sowmya R. Krishnan:** Methodology, Validation, Formal analysis, Visualization, Writing – review & editing. **Yasien Sayed:** Conceptualization, Validation, Resources, Writing – review & editing, Supervision. **M. Michael Gromiha:** Conceptualization, Methodology, Validation, Investigation, Resources, Writing – review & editing, Supervision.

## Declaration of competing interest

We state that there is no conflict of interest.

## Data Availability

Data will be made available on request.
