# Supplementary material for: Mechanism of drug resistance in HIV-1 protease subtype C in the presence of Atazanavir
Source: Curr Res Struct Biol. 2024 Feb 20;7:100132. doi: 10.1016/j.crstbi.2024.100132 (PMC10907180; doi:10.1016/j.crstbi.2024.100132)
Supplement: Multimedia component 1 [file mmc1.docx]

**Mechanism of drug resistance in HIV-1 protease subtype C in the presence of Atazanavir**

**S. V. Sankaran^1^, Sowmya R. Krishnan^1^, Yasien Sayed^2^ and M. Michael Gromiha^1,*^**

^1^Department of Biotechnology, Bhupat and Jyoti Mehta School of Biosciences, Indian Institute of Technology Madras, Chennai 600036, India

^2^Protein Structure-Function Research Unit, School of Molecular and Cell Biology, University of the Witwatersrand, Johannesburg, South Africa

**
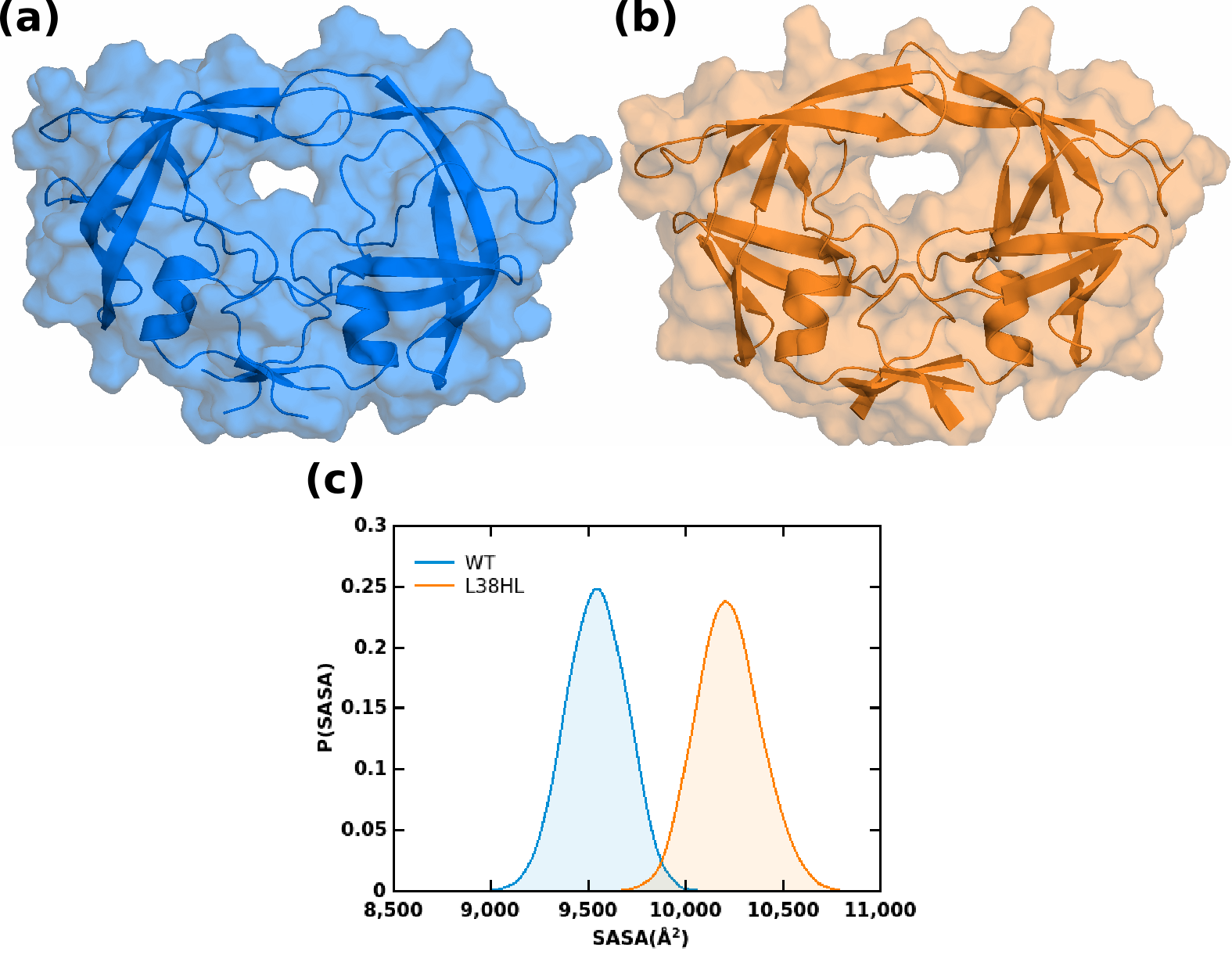
Supplementary information**

**Figure S1** Representative 3D structures for comparing the change in active site volume. Both cartoon and surface representations are used for visual clarity. (a) WT, (b) L38HL. (c) The average distribution of solvent-accessible surface area

**
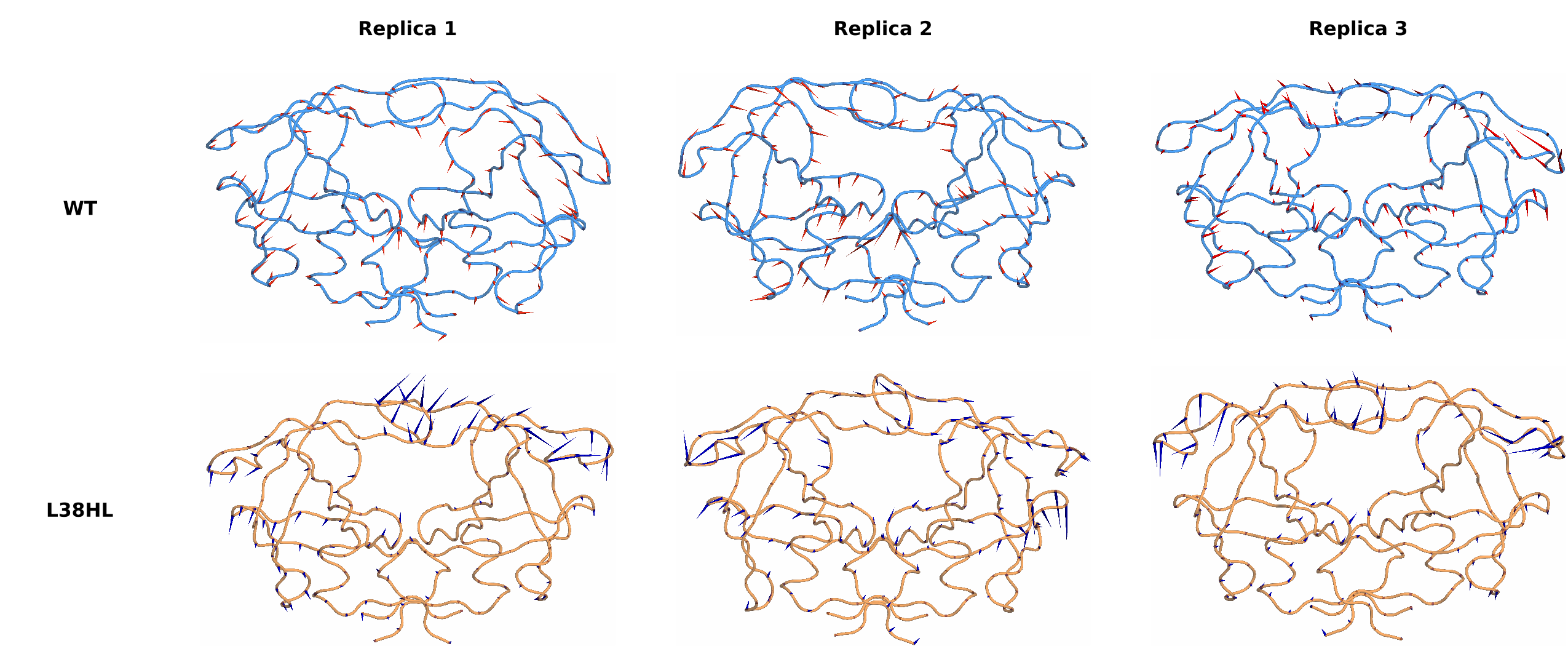
**

**Figure S2** Porcupine plot from essential dynamics (ED) analysis

**
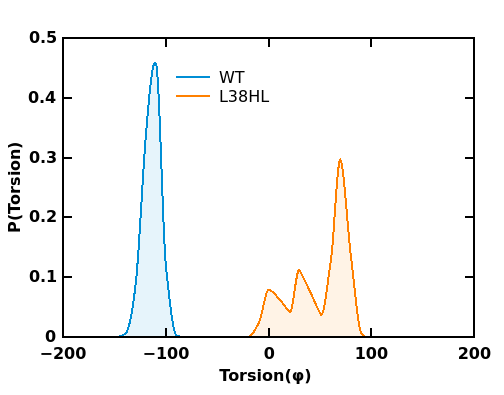
(a)** **(b)**


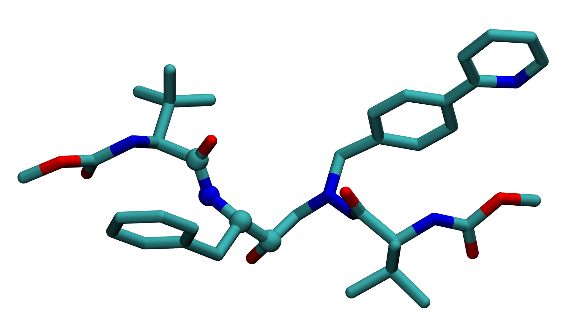


**CBS**

**CBT**

**NBG**

**C**

**Figure S3** (a) Average distribution of ligand dihedral angle. (b) Representative ATV structure depicting the atoms used for dihedral calculation
